# Prevalence and severity of alopecia lesions on gray bats, *Myotis grisescens*, peaks during lactation

**DOI:** 10.1371/journal.pone.0314009

**Published:** 2024-12-02

**Authors:** Ashleigh B. Cable, Megan Kinsella, Richard Gerhold, Elizabeth Hamrick, Cory Holliday, Chris Ogle, Robert T. Stinson II, Dustin Thames, Emma V. Willcox

**Affiliations:** 1 School of Natural Resources, University of Tennessee, Knoxville, TN, United States of America; 2 College of Veterinary Medicine, University of Tennessee, Knoxville, TN, United States of America; 3 Tennessee Valley Authority, Knoxville, TN, United States of America; 4 Tennessee Wildlife Resource Agency, Morristown, TN, United States of America; 5 The Nature Conservancy, Tennessee Chapter, Granville, TN, United States of America; 6 Tennessee Wildlife Resource Agency, Nashville, TN, United States of America; Institut Pasteur de Madagascar, MADAGASCAR

## Abstract

We observed multiple gray bats (*Myotis grisescens*) in 2022 with large patches of fur loss (i.e., alopecia) on the dorsal surface of their body. Alopecia in wildlife has been linked to multiple possible factors and often is a sign of suboptimal health. In 2023, we designed an experiment to compare prevalence of alopecia in gray bats across various reproductive stages, characterize the severity of alopecia lesions, and determine the ectoparasites and microbiota present on the regions of fur loss. We harp-trapped four summer gray bat roosts 2–3 times between 11 April–30 August 2023 and collected skin swabs and scrapes from each bat with alopecia. We determined the severity of the alopecia lesions on a scale 0–7 by summing the degree of redness (0–2), skin condition (0–1), and percentage of fur loss (0–4). We cultured the skin swabs for fungal and bacterial growth and examined skin scrapes under a microscope to determine the presence of subcutaneous mites. We found no evidence that subcutaneous mites cause the fur loss. We determined that prevalence of alopecia in *M*. *grisescens* varies throughout the summer. Prevalence is highest for female bats that are or recently were lactating, reaching an average of 6% ± 6 SD (0–15% range) of captured females exhibiting fur loss during the pup rearing period. Alopecia is most prevalent in male bats in early summer (1% ± 2 SD; 0–4% range). Lactating females had more severe cases than males and were often associated with skin redness due to unknown causes. Bats with alopecia did not differ in body condition, determined from body mass, from bats without alopecia. Future studies could investigate the role of stress in possible autoimmune responses contributing to alopecia. Conservation strategies aimed at reducing stress and supporting nutritional requirements during the summer are likely beneficial to *M*. *grisescens*.

## Introduction

There is global concern for the health of bat populations as they provide essential ecosystem services [1,2]. Recently, there have been multiple observations published on alopecia in bats across the globe, noting individuals of various species with substantial fur loss [3–5]. Alopecia in wildlife has numerous possible causes and has been associated with reproductive condition [6], malnutrition [7], ectoparasites, bacterial and fungal pathogens [8], and more. To date there have been approximately 41 bat species in 5 families documented with the phenomenon [3,4], with the incidence of published observations increasing within the last 2 decades. One concern is that alopecia can be a sign of stress and associated with suboptimal health [3,4,7,9], including nutritional deficiencies, stress, hormonal imbalance, illness, and skin disorders in humans [10]. In bats, causes have been linked to similar factors including toxicants, parasites, endocrine functions, and anthropogenic stressors [3,11]. Most often, observations are sex-biased, with more observations in lactating female bats than male bats, and related to reproductive processes [3]. In the neotropics, alopecia in frugivorous species of bats can be related to climate, with most observations occurring in the dry season. Additionally, the same study determined that degree of urbanization could influence prevalence [11]. Alopecia has not been associated with mortality in bats. However, considering global climate change and many other emerging threats to bats, understanding the sublethal impacts and underlying causes of alopecia are important for population monitoring.

*Myotis grisescens*, commonly the gray bat or gray myotis, is a cave-hibernating bat species that occupies karst areas of the Southeast USA. They are a federally endangered species of longtime conservation interest due to their declining populations in response to habitat disturbance [12]. They hibernate in massive colonies—up to 350,000 bats or more—in the winter and migrate to form large maternity colonies in the summer to rear pups. *Myotis grisescens* are a known host for many parasites and their communal roosting strategy leads to high parasitic loads of mites [13]. While they are often observed with mites, there are no known negative impacts to bat health from the documented parasites. *Myotis grisescens* are sympatric with many bat species that are affected by a non-native fungal pathogen (*Pseudogymnoascus destructans*) that can cause white-nose syndrome; however, while the causal fungus has been detected on *M*. *grisescens*, the species does not suffer mass-mortality from the disease like some other species. Cave disturbance remains the most substantiated threat to date [12].

In 2022, researchers in Tennessee, USA made observations at mist-net sites and caves of *M*. *grisescens* with bald patches and lesions on the skin above the scapulae, which in some cases extended across the dorsum and head. The observations of alopecia in *M*. *grisescens* were noted mostly on reproductively active (i.e., lactating and post-lactating) adult females, except for two milder cases on males. Trapping events that occurred later in the summer yielded no observations of alopecia on males or females [14]. Noting these observations, we designed an experiment to 1) quantify the prevalence of alopecia in *M*. *grisescens* at summer roosts, 2) determine if prevalence is related to reproductive processes (i.e., the high energy demands of lactation), 3) investigate the relationship of alopecia with body condition, 4) classify the severity of the lesions, and 5) identify the ectoparasites and microbiota (i.e., bacteria and fungi) associated with the lesions. Given our previous observations and those of other studies [6], we hypothesized that alopecia prevalence would be highest when female bats produce milk and raise young as there might be a trade-off between energy required for fur growth and reproduction. This hypothesis is supported by reports of hair and fur loss in studies of human and non-human mammals due to an increase in the percentage of hairs that remain in the non-growing stage (i.e., telogen phase) during pregnancy, thus, leading to excessive post-partum shedding [15,16]. Given that, in many other taxa and bats species, alopecia is sometimes caused by ectoparasites or pathogenic [8,9,17–19], we also hypothesized that lesions would be accompanied by the presence of skin mites or certain bacteria or fungi known to cause fur loss in other organisms. Additionally, we hypothesized that bats with alopecia might have nutritional deficiencies causing the condition, thus we tested if bats with alopecia would have lower body masses than bats without it, as this has been seen in polar bears [20].

## Materials and methods

### Study area and species

**Fig 1:**
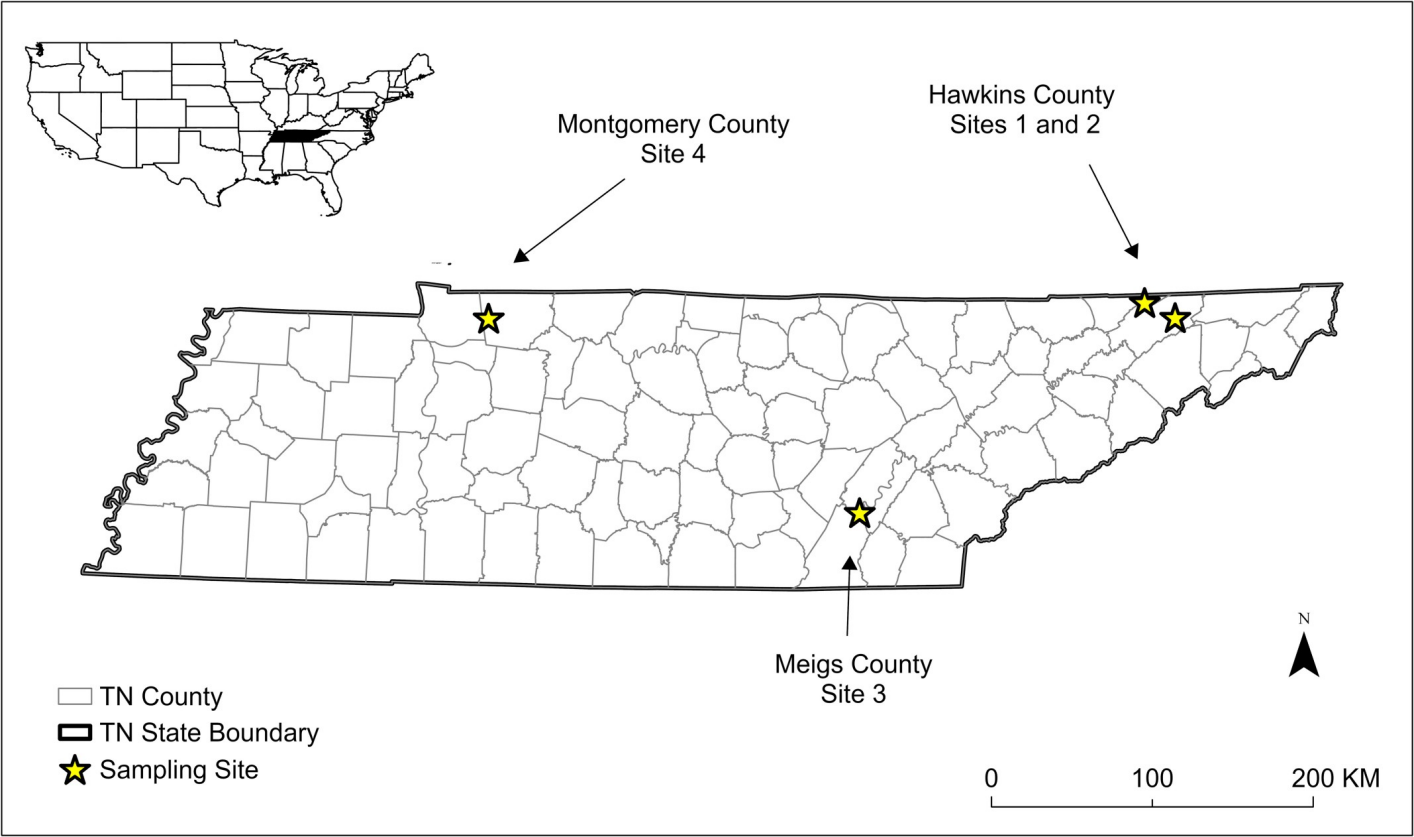
Map of four sampling sites where bats were captured in Tennessee (TN), USA in summer 2023 to investigate prevalence and possible causes of alopecia. Geopolitical boundaries are provided by the United States Census Bureau.

We conducted harp-trap (Bat Conservation and Management, Inc.) surveys to capture bats at four summer roosting sites in Tennessee, (TN), USA: a cave in Hawkins County (Site 1; 632 m from closest major road with 2 cars or more/minute daily traffic; 0% urban development in surrounding 500 m radius), an unfinished concrete nuclear facility in Hawkins County (Site 2; 3,584 m from closest major road; 24% urban development in surrounding 500 m radius), a cave in Meigs County (Site 3; 583 m from closest major road; 0% urban development in surrounding 500 m radius), and a cave in Montgomery County (Site 4; 5,063 m from closest major road; 0% urban development in surrounding 500 m radius) (Fig 1). Distance to closest major road and urbanization metrics were extracted from Geographic Information System layers created from a previous study [21]. Sites 1 and 4 are heavily used by *M*. *grisescens* in the summer and winter and Sites 2 and 3 are known to be primarily used during the summer season. *Myotis grisescens* make large movements from caves between the summer and winter [22]. Many move to different caves to form large maternity colonies to raise pups. They also form bachelor colonies composed of males and some summer sites have mixed colonies of males and females. For these reasons, we chose these sites to get a large sample size from both sexes. We did not collect climate data inside the caves or nuclear facility; however, all sites were in TN, USA, and temperature means for June 2023 (mid-summer) were similar for Site 1 (20°C), Site 2 (20°C), and Site 3 (22°C). Site 4 was in a county with slightly warmer temperatures (23.5°C). Average precipitation in June 2023 was 10.8 cm for Sites 1 and 2, 12.9 cm for Site 3, and 5.1 cm for Site 4 (National Oceanic and Atmospheric Administration data found at https://www.ncei.noaa.gov/access/monitoring/climate-at-a-glance/county/mapping/40/pcp/202306/1/value).

### Bat capture

All bat handling and sample collection followed white-nose syndrome decontamination protocols, was approved by the University of Tennessee IACUC committee (Protocol 2253) and was performed under state and federal research permits (ES11170C–3 to ABC, TE35313B to EVW). We sampled Site 1, Site 2 and Site 3 three times throughout the summer and sampled Site 4 twice. The sampling times were selected to target bats during the pre-pregnancy or early pregnancy time and when males are typically non-reproductive (sampling round 1: April 11–25), during or soon after female lactation (round 2: July 5–10), and the time before migration when females are no longer lactating and males are in reproductive condition (i.e., testes are descended) for fall mating (round 3: August 25–30). Site 4 was only sampled during round 1 and 2 to limit disturbance, as it is a major summer and winter hibernacula for the species. We also limited the numbers of captures during sampling round 1 and 2 at Site 4 for these reasons. We limited the captures to 30 bats for sampling round 1. The target numbers were increased to 100 bats for round 2 as the sampling event occurred during a simultaneous research project that required more captures. For Sites 1–3, we did not limit the number of bats we sampled. However, we would move the harp trap away from the entrance periodically throughout each sampling event to avoid an excess of bats waiting to be processed, as specified in our USFWS federal recovery permit that limits holding times.

For each bat that was captured, we recorded morphometric and demographic data, including species, age, sex, right forearm length (mm), mass (g), presence of alopecia (yes/no), and any observational notes. We recorded reproductive condition as non-reproductive (females were not pregnant or lactating and males did not have descended testes), pregnant (females had rounded abdomen), lactating (females had localized fur loss and swelling around the nipple, had signs of the young chewing on the nipple, or had milk expressed from the nipple), post-lactating (females had recent signs of chewing on the nipple or fur regrowth around the nipple), or males with testes descended. Observational notes included any bat recaptures or any wing or other injuries. We determined if a bat was a recapture by marking individuals at each cave with 2.9 mm aluminum lipped bands with a unique identifier (Porzana, UK) and applied by trained personnel using banding pliers to ensure a circular fit with no crimped edges and a standardized gap size in the open end of the band. While some bat species may not tolerate bands well [23], mark-recapture studies using lipped bands has been used on *M*. *grisescens* for decades to study various aspects of their ecology with minimal reported observations of harm [12,22,24–26] and is an approved method by IACUC and our USFWS federal recovery permits. We did not mark juvenile bats that weighed less than 8 grams. This was an arbitrary decision the authors made to reduce stress on newly volant juvenile bats and was not based on any previous recommendation. However, there were no juveniles observed with alopecia in this study and we did not recapture any of the bats that we marked, thus, this decision likely did not influence comparisons of prevalence across the 3 sampling rounds.

### Prevalence of alopecia lesions and possible nutritional deficiencies

We first determined the overall prevalence of alopecia by calculating the percentage of *M*. *grisescens* that we encountered with alopecia from the total captured. We then calculated the percentage with alopecia by sampling round, sex, and reproductive condition. We used a Fisher exact test and a pairwise Fisher post hoc test with a Bonferroni correction for multiple comparisons to determine if prevalence varied by season (i.e., sampling round). We used Chi-square tests to determine if prevalence varied by sex. Then we used a Fisher exact test with pairwise comparisons for post-hoc tests with a Bonferroni correction for multiple comparisons to determine if prevalence varied by reproductive condition. We used data from lactating and post-lactating females only to determine if bats with alopecia differed in body condition, a potential indicator of nutritional deficiencies. We used a Kruskal-Wallis test to determine if mass differed in lactating and post-lactating females with alopecia versus without. We chose only the reproductively active females because they had the highest prevalence of alopecia (discussed in results) and reproductive processes can influence body mass.

### Severity of alopecia lesions and associated ectoparasites and Microbiota

To determine severity of alopecia, we classified each case into 3 categories based on a scoring rubric. First, we scored based on the degree of erythema (i.e., redness). If the affected skin was mostly red and irritated then we scored the lesion as a 2, if it was mildly red or pink, we scored it as a 1, and if it was not red at all then we scored it as a 0. Second, if the skin was flaky, scaly, bumpy, or crusty then it was scored as a 1 and if it was smooth then it was a 0. Lastly, we ranked the size of the lesion on a scale 0–4 by estimating the percentage of the entire dorsal surface of the bat that was missing fur. The size of the lesion was 1 if the region of fur loss was between 1–24%, 2 if between 25–49%, 3 if between 50–74%, and 4 if between 75–100%. The overall severity scores based on this rubric could range between 1–7 for bats with alopecia.

To determine if the condition was related to an ectoparasite causing sarcoptic mange, we used a size 10 scalpel coated in mineral oil to perform a skin scrape to collect skin mites on the area of fur loss for each alopecia bat. We transferred the scalpel to a sterile tube, transferred it to the lab, and prepared slides to examine under a compound microscope using the 4X and 10X objective lenses. We consulted an expert at the University of Tennessee Veterinary Medical Center to identify any mites found. We only did this for a single mite.

We used bacteriology swabs and plucked fur around the periphery of the affected area on the bat to classify the associated microbiota on the lesions. Swabs were stored in Amies gel and fur was stored in an empty sterile vial and were submitted to the Bacteriology Lab at the College of Veterinary Medicine at the University of Tennessee for aerobic and fungal culture. The swabs and fur samples were used to inoculate a Columbia blood agar plate with 5% sheep blood, a Columbia Nalidixic Acid agar plate with 5% sheep blood, and a MacConkey II agar plate for aerobic culture using a four-quadrant streaking isolation method. Hair samples were placed on split agar plates with dermatophyte test medium and Sabouraud dextrose with brain heart infusion agar.

Aerobic culture plates were incubated at 37°C with 5% CO^2^ for blood containing media and 35°C ambient air for MacConkey and checked daily for growth for 5 days. Each colony type was recorded, sub-cultured, and identified using Gram stain and Matrix Assisted Laser Desorption Ionization-Time of Flight (MALDI-TOF). A catalase test was performed on isolates that were unable to be identified using MALDI-TOF; catalase positive Gram-positive cocci were categorized as *Staphylococcus*, and catalase negative Gram-positive cocci were categorized as resembling *Streptococcus* or *Enterococcus*.

Fungal cultures were incubated at room temperature in ambient air and held for five weeks and checked weekly. Each fungal colony was subcultured to Inhibitory Mold agar and phenotypically identified microscopically using a tape prep with lactophenol aniline blue stain according to Larone’s Medically Important Fungi. Fungal identification was also attempted using MALDI-TOF.

## Results

### Prevalence of alopecia lesions and possible nutritional deficiencies

**Fig 2:**
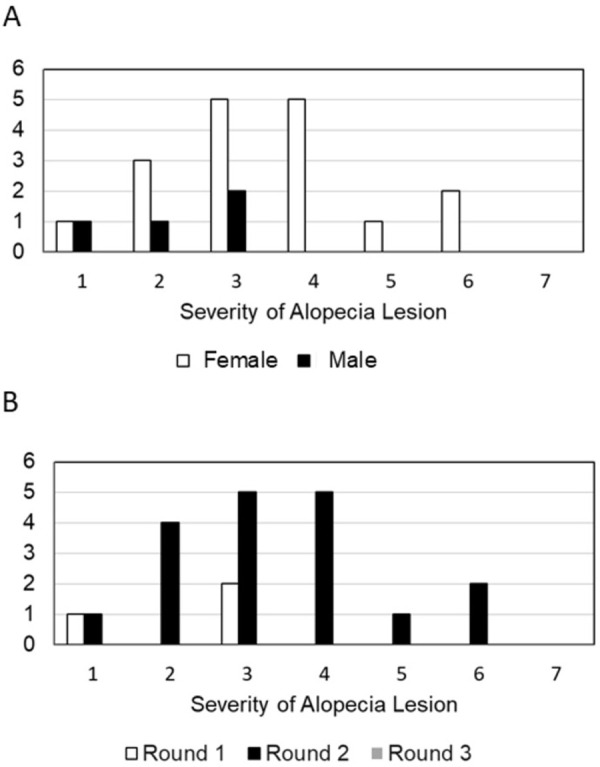
Severity of alopecia lesions by sex (Fig 2A) and sampling round (Fig 2B) from bats captured in the Tennessee, USA in 2023. Sampling round 1 occurred in late spring/early summer (sampling round 1: April 11–25), in mid-summer (round 2: July 5–10), and in late summer/early fall (round 3: August 25–30). Severity of each alopecia lesion was calculated using a rubric incorporating redness, skin condition, and percentage of fur loss on the dorsum. The severity ranged on a scale 0–7, with 7 being the most severe lesion.

We captured 1,179 total *M*. *grisescens* (n = 489 females, n = 690 males) in this study. Overall prevalence of alopecia in the sampled population was 1.9%. However, prevalence varied by sex and sampling round (Table 1) (Fig 2). Prevalence was highest for females during the second sampling round (6.3% ± 6.4 SD), coinciding with lactation and post-lactation. Prevalence was highest for males during the first sampling round (1.0% ± 2.1 SD), the time following spring migration. Prevalence for females during this same period following migration was higher than males at 1.6% ± 1.5 SD. The Chi-square test revealed that occurrence of alopecia significantly varied between males and females (χ2 = 10.758, df = 1, p = 0.001). Reproductive condition was significant in bats with alopecia versus without (p = 2.17^e-09^) and the significance was driven by higher prevalence in female lactating and post-lactating bats compared to pregnant females, non-reproductive males and females, and reproductively active males (i.e., males with testes descended).

**Table 1:** Prevalence of gray bats (*Myotis grisescens*) captured with alopecia from sites in Tennessee, USA. Sampling round 1 occurred in late spring/early summer (April 11–25), sampling round 2 occurred mid-summer (July 5–10), and round 3 occurred late summer/early fall (August 25–30). Sites were four summer roost sites: A cave in Hawkins County (Site 1), an unfinished concrete nuclear facility in Hawkins County (Site 2), a cave in Meigs County (Site 3), and a cave in Montgomery County (Site 4).

Sampling Round	Site	Bats captured (n)	Bats with alopecia (% (n))	Females captured (n)	Females with alopecia (% (n))	Males captured (n)	Males with alopecia (% (n))
Sampling Round 1	1	222	0.9 (2)	62	3.2 (2)	160	0.0 (0)
	2	49	2.0 (1)	18	5.6 (1)	31	0.0 (0)
	3	11	0.0 (0)	5	0.0 (0)	6	0.0 (0)
	4	29	3.4 (1)	5	0.0 (0)	24	4.2 (1)
Avg		78	1.6 (1)	23	2.2 (1)	55	1.0 (0)
SD		97	1.5 (1)	27	2.7 (1)	71	2.1 (1)
Sampling Round 2	1	184	1.6 (3)	18	0.0 (0)	166	1.8 (3)
	2	183	4.9 (9)	121	7.4 (9)	62	0.0 (0)
	3	59	1.7 (1)	32	3.1 (1)	27	0.0 (0)
	4	100	5.0 (5)	27	14.8 (4)	73	1.4 (1)
Avg		132	3.3 (5)	50	6.3 (4)	82	0.8 (1)
SD		62	1.9 (3)	48	6.4 (4)	59	0.9 (1)
Sampling Round 3	1	74	0.0 (0)	24	0.0 (0)	50	0.0 (0)
	2	172	0.0 (0)	89	0.0 (0)	83	0.0 (0)
	3	96	0.0 (0)	88	0.0 (0)	8	0.0 (0)
Avg		114	0.0 (0)	67	0.0 (0)	47	0.0 (0)
SD		51	0.0 (0)	37	0.0 (0)	38	0.0 (0)
Total		1,179	1.9 (22)	489	3.5 (17)	690	0.7 (5)

The average body mass for females during the period of the highest alopecia prevalence was 10.4 g ± 1.0 SD for those with the condition and 10.3g ± 0.8 SD for those without. Body condition was not significantly different between lactating and post-lactating females with alopecia compared to those without the condition (χ2 = 0.059452, df = 1, p = 0.807). See supplemental table for photos of bats with alopecia and severity scores (S1 Table).

### Severity of alopecia lesions and associated ectoparasites and microbiota

We collected 26 skin scrapes (n = 21 alopecia samples, n = 5 reference samples) and counted a single mite on 1 bat without alopecia. The mite was identified as resembling *Chiroptoglyphus americanus*. We cultured samples from 24 bats (n = 21 alopecia samples, n = 3 reference samples). The reference samples were swabs collected from 1 bat without alopecia from each site that we sampled, excluding Site 4. We detected 56 identifiable bacteria and fungi species and 7 unknown species on alopecia samples. We detected 9 bacteria and fungi species on reference samples. Various species of *Staphylococcus* bacteria were common on both alopecia and reference bat samples. There were multiple bacteria and fungi present on alopecia lesions (Table 2). The bacteria and fungi present on the two most severe lesions exhibiting erythema and larger areas of fur loss were *Staphylococcus sciuri*, *Staphylococcus cohnii*, *Staphylococcus lentus*, *Staphylococcus nepalensis*, *Penicillium* spp., and *Cladosporium* spp. All of these were also detected on reference bats, except for *Cladosporium*. However, *Cladosporium* was only detected in 2 of the 21 alopecia samples.

**Table 2:** The microbiota associated with alopecia and healthy skin of bats captured in summer 2023 in Tennessee, USA listed in order of lesion severity.

Bat ID	Redness (0–2)	Skin condition (0–1)	Size (1–4)	Severity (0–7)	Microbiota present
MYGR 5154	2	1	3	6	*Staphylococcus sciuri*, *Staphylococcus cohnii*, *Penicillium* spp.
MYGR 5162	2	0	4	6	*Staphylococcus lentus*, *Staphylococcus nepalensis*, *Cladosporium* spp.,
MYGR 5323	0	1	4	5	*Staphylococcus aureus*, *Staphylococcus* spp., no fungal growth
MYGR 5152	1	1	2	4	*Staphylococcus spp*., *Staphylococcus lentus*, *Enterococcus faecalis*, *Chryseobacterium shandongense*, *Staphylococcus cohnii*, unknown fungal spp.
MYGR 5157	2	0	2	4	*Staphylococcus nepalensis*, unknown bacteria spp., *Alternaria* spp.
MYGR 5158	1	0	3	4	*Staphylococcus lentus*, *Staphylococcus nepalensis*, *Cladosporium* spp., no fungal growth
MYGR 5159	1	1	2	4	*Staphylococcus nepalensis*, unknown gram-positive bacteria
MYGR 5428	0	0	4	4	No sample taken
MYGR 4662	1	0	2	3	*Staphylococcus* spp., *Macrococcus canis*, *Scopulariopsis* spp.
MYGR 4676	0	1	2	3	*Staphylococcus spp*., *Psychrobacter* spp., *Aspergillus* spp., unknown fungal spp.
MYGR 5156	0	1	2	3	*Staphylococcus lentus*, *Staphylococcus nepalensis*, *Staphylococcus sciuri*, no fungal growth
MYGR 5161	1	0	2	3	*Staphylococcus lentus*, *Penicillium* spp.
MYGR 5167	0	1	2	3	*Staphylococcus cohnii*, *Staphylococcus succinus*, *Penicillium citrinum*
MYGR 5170	1	0	2	3	*Staphylococcus cohnii*, *Staphylococcus sciuri*, *Penicillium* spp., *Purpureocillium lilacinum*
MYGR 5312	1	1	1	3	No bacterial or fungal growth
MYGR 5074	0	0	2	2	No bacterial or fungal growth
MYGR 5163	0	1	1	2	*Staphylococcus lentus*, *Corynebacterium bovis*, *Staphylococcus* spp., *Aspergillus* spp.
MYGR 5310	1	0	1	2	*Staphylococcus cohnii*, *Corynebacterium* spp., *Purpureocillium lilacinum*, *Penicillium* spp.
MYGR 5318	1	0	1	2	No bacterial or fungal growth
MYGR 4749	0	0	1	1	*Staphylococcus lentus*, unknown gram-positive bacteria spp., *Penicillium* spp., *Scopulariopsis* spp.
MYGR 5322	0	0	1	1	*Staphylococcus* spp., no fungal growth
MYGR 5104 (no alopecia)	0	0	0	0	No bacterial or fungal growth
MYGR 5160 (no alopecia)	0	0	0	0	*Staphylococcus sciuri*, *Staphylococcus nepalensis*, *Staphylococcus cohnii*
MYGR 5303 (no alopecia)	0	0	0	0	*Staphylococcus cohnii*, *Staphylococcus saprophyticus*, *Staphylococcus sciuri*, *Purpureocillium lilacinum*, *Penicillium* spp.

## Discussion

We hypothesized that alopecia would be most prevalent in reproductively active female bats, and this was supported by our results. While we only studied one bat species, this appears to be the case in other bat species occurring in North America and globally [3]. Alopecia was present on 44% of bats in a maternity colony of Rafinesque big-eared bats (*Corynorhinus rafinesquii*) in the year 2000, and observations of the phenomenon were recorded again in 2004 and 2011 [5]. Pond bats in the Netherlands exhibit the same pattern of alopecia (i.e., fur loss between the shoulder blades) and prevalence is highest during the female nursing period [6]. Higher prevalence of alopecia cases in reproductively active females could possibly indicate compounding stressors or nutritional deficiencies resulting from a higher energy demand. For example, a lactating little brown bat (*Myotis lucifugus*) requires consumption of a larger amount of insects during lactation [27]. It is well understood that nutritional requirements are substantially higher to support reproductive processes such as fetal development, egg or milk production, maternal care of young, changes in endocrine functions and morphological changes of reproductive organs, and more in wildlife [28]. Studies of captive wildlife show that improved diets can lead to thicker fur and skin condition in animals with deficiencies [29]. *Arctocephalus pusillus doriferus* (Australian fur seals) sampled from the wild that have lower zinc and tyrosine levels are more likely to exhibit fur loss [30]. Thus, improving the foraging areas and diets of bats could improve prevalence of alopecia. Moreover, conservation and recovery of *M*. *grisescens* is often concentrated on fulfilling the summer habitat needs during reproduction and protecting maternity colonies [12]. Thus, understanding the trends associated with females of the species could be critical for population recovery and indicative of underlying issues that could be addressed in conservation planning.

A study in the neotropics determined that alopecia prevalence is tied to factors related to urbanization and climate [11]. The four sites that we sampled were mostly in remote areas of the state with little to no urbanization in the immediate surrounding landscape and over 0.5 km or more away from any major roads. The one site that had the most urbanization (25% in the surrounding 500 m radius), the unfinished nuclear facility (Site 2), did have substantial observations of alopecia; however, this was exceeded at a site with 0% urbanization (Site 4). The influence of urbanization on alopecia in bats on a larger scale at sites with more variation in the degree of urbanization than those considered in this study could be evaluated in future studies. Moreover, the influence of climate variables could be investigated further. The site with the highest observed prevalence of alopecia (Site 4) was in the area that received slightly warmer June temperatures (3.5°C warmer than Sites 1 and 2 and 1.5°C warmer than Site 3). Anecdotally, the area that Site 4 is in also tends to experience more tornados and severe weather events than eastern parts of TN, which could be additional factors contributing to stress-induced fur loss.

There are still many questions to be investigated about the phenomenon of alopecia. It does not appear to be obviously related to molting. For example, alopecia was highest in lactating and post-lactating bats, but bats in this group without alopecia showed no signs of fur loss or thinning. If it was a species trait molt pattern, we might expect to see it in all or most bats within a demographic group. Moreover, there was no new fur growth on alopecia spots during the second sampling round, but we did observe it during sampling round 3. Researchers have suggested that the phenomenon occurring with *Myotis dasycneme* (pond bats) could be related to olfactory cues during reproduction [6]. Certainly, researchers are still making new discoveries about bat use of olfaction. For example, males of *Leptonycteris yerbabuenae* (lesser long-nosed bat) sometimes exhibit a dorsal patch in the interscapular region that they apply bodily fluids to with their feet to attract mates [31]. Given that prevalence is highest in females during lactation, the need to attract a mate is not a likely explanation; however, there could be other reasons to use olfaction if the bald spots excreted some hormone or scent. On the other hand, if the patches were for some olfactory reason, we might expect to see higher prevalence of fur loss in the lactating population. Another common cause of alopecia in wildlife is mange, which is accompanied by various microscopic mites in skin and hair follicles. Evidence of ectoparasite-associated alopecia was not found in any of the sampled bats.

We did not measure cortisol levels, so we cannot compare the amount of stress hormones in bats with alopecia to those without. However, stress hormones peak during pregnancy and lactation in other bat species [32], so it is possible that alopecia prevalence is highest during a time of reproductive stress. Stress hormones have been related to alopecia in captive primates [33] and alopecia sometimes occurs in low-ranking individuals experiencing more resource competition or emotional stress [34]. In one study of primates, hair cortisol and alopecia were both significantly higher in nursing females compared to pregnant females, though there was no correlation between cortisol and alopecia [15]. However, additional studies could look at cortisol levels in bats with and without alopecia to determine if there is a relationship.

There was no obvious microbiota occurring on all alopecia bats. We cannot conclude that bacteria and fungi caused the alopecia in this study. However, the more severe lesions were associated with multiple bacteria and fungi that could possibly manifest as opportunistic skin infections or just be a part of the normal skin microbiome. Some infectious dermatological diseases in *Odocoileus virginianus* are often associated with multiple bacteria and fungi species [8] and this could be explored further for bats.

Despite a substantial prevalence of fur loss in bats mid-summer, there was no significant association with negative body condition. Telogen effluvium alopecia, caused by an increase of hair follicles in the non-growing stage during pregnancy leading to excessive shedding post-partum, as seen in primates [15,16,35], may be a plausible explanation for this phenomenon in this species. This was consistent with our results as prevalence was zero and we observed evidence of new fur growth on captured bats by the end of summer. Our results complement those of a study on *Myotis dasycneme*, where fur regrowth over bald spots occurs following the peak of the nursing period [6]. Therefore, it is likely that it is not an issue substantially impacting populations currently. However, with emerging threats and compounding stressors, monitoring alopecia prevalence in bats captured in the future could be very important if indicative of underlying issues potentially affecting populations. Healthy bat populations are critical to maintain healthy ecosystems and humans [2,36–41]. With increasing pressures on bats, conservation efforts that aim to reduce stress, support nutritional requirements, and monitor climate and land use impacts on bats during reproduction and other vulnerable life stages are likely crucial to maintain healthy global bat populations.

### Dedication

The authors dedicate this manuscript to Chester O. Martin, who passed away in 2023. He contributed to bat conservation through his research and his art works. His last publication was on alopecia in a Rafinesque big-eared bat maternity colony.

## Supporting information

S1 Table Excel file photo log and severity scoring rubric for bats captured with alopecia. (XLSX)
